# Dissolved Organic Carbon Source Attribution in the Changjiang Outflow Region of the East China Sea

**DOI:** 10.3390/s21248450

**Published:** 2021-12-17

**Authors:** Xiaoyu Zhang, Yong Du, Zhihua Mao, Lei Bi, Jianyu Chen, Haiyan Jin, Shuchang Ma

**Affiliations:** 1 School of Earth Sciences, Zhejiang University, Hangzhou 310027, China; bilei@zju.edu.cn (L.B.); 21938046@zju.edu.cn (S.M.); 2 Ocean Academy, Zhejiang University, Zhoushan 316000, China; 3 Hainan Institute, Zhejiang University, Sanya 572000, China; 4 Jiyang College, Zhejiang A&F University, Zhuji 311800, China; duyong@zafu.edu.cn; 5 State Key Laboratory of Satellite Ocean Environment Dynamics, Second Institute of Oceanography, Ministry of National Resources, Hangzhou 310012, China; mao@sio.org.cn (Z.M.); chenjianyu@sio.org.cn (J.C.); 6 Laboratory of Marine Ecosystem and Biogeochemistry, The Second Institute of Oceanography, Ministry of National Resources, Hangzhou 310012, China; jinhaiyan@sio.org.cn

**Keywords:** Changjiang outflow region, chromophoric dissolved organic matter, optical properties, dissolved organic carbon, relationships

## Abstract

The variable optical properties of chromophoric dissolved organic matter (CDOM) under the complicated dynamic marine environment make it difficult to establish a robust inversion algorithm for quantifying the dissolved organic carbon (DOC). To better understand the main factors affecting the relationship between the DOC and the CDOM when the Changjiang diluted water (CDW) interacts with the marine currents on the wide continental shelf, we measured the DOC concentration, the absorption, and the fluorescence spectra of the CDOM along the main axis and the northern boundary of the CDW. The sources of DOC and their impacts on the relationship between the optical properties of the DOC and CDOM are discussed. We reached the following conclusions: There are strong positive correlations between the absorptive and fluorescent properties of the DOC and the CDOM as a whole. The dilution of the terrestrial DOC carried by the CDW through mixing with saline sea water is the dominant mechanism controlling the characteristics of the optical properties of the CDOM. CDOM optical properties can be adopted to establish inversion models in retrieving DOC in Changjiang River Estuary. It is concluded that the introduction of extra DOC from different sources is the main factor causing the regional optical complexity leading to the bias of DOC estimation rather than removal mechanism. As whole, the input of polluted water from Huangpujiang River with abnormally high a(355) and Fs(355) will induce the overestimation of DOC. In the main axis of CDW, the impact from autochthonous DOC input to the correlation between DOC and CDOM can be neglected in comparison with conservative dilution procedure. The relationship between the DOC and the CDOM on the northern boundary of the CDW is more complicated, which can be attributed to the continuous input of terrestrial material from the Old Huanghe Delta by the Subei Coastal Current, the input of materials from the Yellow sea by the Yellow Sea Warm Western Coastal Current, and the input of materials from the Changjiang Basin by the CDW. The results of this study suggest that long-term observations of the regional variations in the DOM inputs from multiple sources in the interior of the CDW are essential, which is conducive to assess the degree of impact to the DOC estimation through the CDOM in the East China Sea.

## 1. Introduction

As the third longest river in the world, the Changjiang River delivers 0.5–0.8% of the riverine dissolved organic carbon (DOC) to the global oceans [1], exerting a significant influence on the carbon budget and the marine eco-environment of the East China Sea (ECS) and the western Pacific Ocean. The accurate estimation of the DOC flux into the ECS and its temporal variations is critical to determining whether the Changjiang Estuary is a carbon sink or a carbon source, and to clarifying its significance to the global carbon balance. In the past few decades, in order to obtain the DOC content using satellite remote sensing with large-scale coverage and real-time monitoring capabilities, significant efforts have been devoted to establishing inversion algorithms for the DOC based on the optical properties of the chromophoric dissolved organic matter (CDOM). However, to date, no feasible algorithm has been developed that is universally valid and applicable to the Changjiang Estuary. The correlation between the CDOM and the DOC in the Changjiang Estuary has been reported to vary significantly depending on the location, spatial scale, season, and tides [2,3,4,5]. The variable DOC composition has been concluded to be one of the main factors impairing the stability of the relationship between the DOC and the CDOM [6,7]. For example, the mutual linear correlation between the DOC and the CDOM could easily collapse in places with significant phytoplankton production [6,8]. Furthermore, the nonconservative behavior of the DOC is related to the abnormal introduction of DOC into or removal of DOC from the estuary and the adjoining sea area. The release of DOC from the pore water during the disturbance or resuspension of the bottom sediments has been demonstrated to be one of the extra DOC inputs, i.e., in addition to the riverine-sourced DOC [8,9]. Recently, sporadic precipitation was reported to be one of the main DOC material sources in coastal sea water [10]. Conversely, microbial degradation [11] and photo bleaching [12,13] are two of the main DOC removal mechanisms. The extra input or removal of DOC not only changes the DOC content, but it also introduces different DOC species and/or selectively removes DOC species, which leads to the variable relationship between the DOC and the CDOM. However, in most studies that have focused on the estuary, more effort was devoted to understanding the impact of the estuarine effects on the conservative behavior of the DOC and to determining the feasibility of an inversion algorithm for determining the DOC from the CDOM [14]. Concerning the amount of terrestrial DOC and its geochemical behaviors on the wide continental shelf when encountering saline oceanic currents and the impacts on the marine ecological system, studies of the changes in the DOC content and species after the Changjiang diluted water (CDW) enters the ECS need to be performed. In addition, due to the continuous decrease in the suspended sediment flux transported by the CDW, the potential sediment compensation via erosion of the submarine delta and from the Old Yellow River Delta [15] may change the composition of the material flowing into the ECS. Therefore, it is valuable to observe the land-source DOC behavior and to identify the main factors affecting its distribution, species, and optical properties over the entire continental shelf during land–sea interactions.

In this study, we collected samples from two sections: the main axis and the northern boundary of the CDW, combining with a section in the southern branch of the Changjiang River as reference. The DOC content, CDOM optical properties, and salinity of each sample were measured. A combination of excitation–emission matrix fluorescence spectroscopy (EEMS) and parallel factor analysis (PARAFAC) was used to determine the compositions and sources of the fluorescent materials in these samples. The relationships between the optical properties of the DOC and the CDOM and the salinity were analyzed using linear regression. Based on the optical properties of the CDOM and their ability to reflect the DOC content and species, we attempted to answer the following three questions. (1) What is the main mechanism determining the relationship between the optical properties of the DOC and the CDOM in the CDW after it travels beyond the mouth of the estuary? (2) What are the main differences in the relationships between the DOC and the CDOM along the main axis and within the northern boundary of the CDW? (3) What type of input or removal procedures cause the differences in the relationships between the DOC and the CDOM along the main axis and within the northern boundary of the CDW?

The results of this study deepen our understanding of the different mechanisms affecting the DOC content, species, and distribution and the intrinsic factors determining the optical properties of the CDOM in the Changjiang outflow region under the conditions of this complicated marine environment, which is essential to establishing a feasible algorithm for regionally inverting the optical properties of the DOC from the CDOM. These results will also help to identify the material sources of the dissolved organic matter (DOM) in this sea area.

## 2. Materials and Methods

### 2.1. Hydrodynamic Environment and Sampling

The ECS is one of the world’s largest marginal seas and contains a wide continental shelf. The spatial and temporal distributions of the substances in the ECS are caused by the comprehensive effects of multiple sources of terrestrial material and complex hydrodynamic oceanic processes. The migration route and magnitude of the CDW determines the transportation, diffusion, and mixing of the materials in the ECS. Usually, the main axis of the CDW migrates southeastward in winter and is variable in summer due to the flood discharge, topographic effects, wind stress, and other factors experienced when encountering the saline Taiwan warm current (TWC). When the TWC intrudes into the inner shelf of the ECS along the 50 m isobaths at the mouth of the Changjiang River, the CDW changes its migration route from southeastward to eastward, or even toward the northeast (around 122.5° E) [16,17]. The large amount of terrestrial sediment transported by the CDW in the bottom layer has developed a huge modern tongue-shaped underwater delta, which extends southeastward. This delta can be disturbed and resuspended, and can have a significant impact on the formation of the maximum turbidity zone (MTZ).

**Figure 1:**
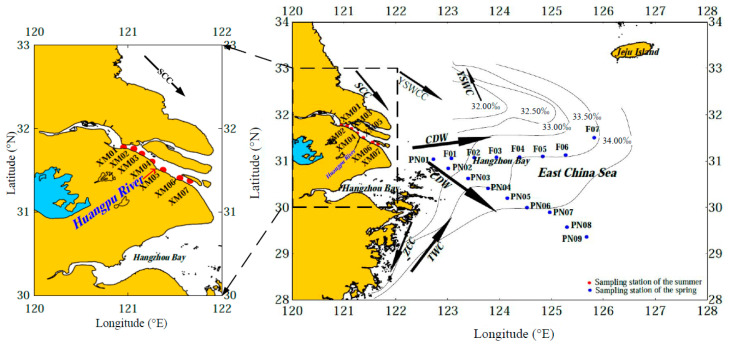
Diagram showing the hydrodynamic environment (in spring) and the sampling stations (modified from [18,19,20]). CDW: Changjiang diluted water; SCC: Subei coastal current; TWC: Taiwan warm current; YSWC: Yellow Sea warm current; YSWCC: Yellow Sea western coastal current; ZCC: Zhejiang coastal current. Section PN begins in the Zhoushan sea area and ends in the Ryukyu Islands, running across the continental shelf of the ECS with water depths ranging from ~100 m to >1000 m. Section PN represents the main axis of the CDW, and it vertically converges with the Kuroshio Current. Section F represents the northern boundary of the CDW, and it is the interface between the ECS and the Yellow Sea. Nine stations (PN01–PN09) with a regular spacing of 42.9 ± 3.3 km were sampled along section PN. Seven stations (F01–F07) with an average spacing of 46.1 ± 9.3 km were sampled along section F. In addition, section XM represents the riverine end member. Seven stations were evenly distributed (with an average spacing of 13.8 ± 3.5 km) along the southern branch of the Changjiang River, which has an oligohaline environment.

In this study, nine water samples were collected along the main axis of the CDW and seven water samples along the northern boundary of the East China Sea (referred to as sections PN and F, respectively, in this study) during the “973” Spring Voyage in 2011 (the stations are marked in blue in Figure 1). Seven water samples from the southern branch of the Changjiang River were collected in August 2011 (referred to as section XM, marked in red in Figure 1).

The fan-like area between the PN and F sections is influenced by the estuarine mixed water (caused by the transition between the fresh CDW and the saline sea water) from either the Subei Coastal Current (SCC), the Yellow Sea Warm Western Coastal Current (YSWCC), and the Yellow Sea Warm Current (YSWC) from the north [14,19] or from the coastal upwelling off the Zhejiang coast [21]. The convergence of these different currents results in a complicated hydrodynamic environment in the area of 123–123°30′ E. A large number of smaller organic particles agglomerate, form larger aggregates, and sink at a faster rate in this area, producing sea snow [20], which inhibits most of the materials transported by the CDW from traveling beyond 123° E. The rich nutrient supply in this area fosters the most important fishing area (i.e., the Zhoushan fishing area) in China (122–130° E, 29–33° N) [22,23]. Due to the scientific significance of the effects of the interaction between the continental shelf and the open ocean water masses on the biogeochemistry and ecology, from the last century onward, continuous long-term observations have been performed in the two sections and in the neighboring sea area by Chinese, Japanese, and Korean researchers to investigate the decadal, interannual, and seasonal variations in the hydrodynamics [24], sedimentation [25], marine ecology, sea water chemistry [26,27], land–sea interactions [28], and particularly, the carbon flux and cycle [29]. These previous studies have provided fruitful and precious information that has deepened our understanding of global climate change [30,31], episodic climate events (e.g., typhoons), and the impact of climate on the development of estuarine hypoxia events [32,33].

The sampling in the PN and F sections was performed during the same period. The first sample in the PN section (22 March 2011) was collected only 2 days after the first sample was collected in the F section (20 March 2011). The longest storage time was 10 days, which is much shorter than the decay half-lives of the different fluorescence components [34], so distinct changes in the absorption intensity due to storage time were minimized [35]. All of the surface seawater samples collected for the DOC and CDOM optical measurements were collected at a water depth of 2 m. At each sampling station, 250 mL aliquot subsamples were filtered using a GF/F filter (47 mmϕ) (combusted at 450 °C for 24 h in a muffle furnace, then prepackaged in clean aluminum foil) and were stored in the dark at −20 °C in precleaned polypropylene narrow-mouth buckets. The frozen samples were thawed and allowed to reach room temperature after the samples were transported to the laboratory. A 60 mL subsample was filtered through a 0.2-μm Nuclepore polycarbonate membrane (soaked in 10% HCl for 15 min and then rinsed with distilled water three times before the filtration) before the CDOM optical measurements. Another 30 mL subsample was used for the DOC concentration measurements. The filtered samples were preserved in 60-mL brown glass bottles, which were precombusted at 450 °C for 6 h in a muffle furnace before use [8,29,36].

### 2.2. Methods

#### 2.2.1. Absorption Spectroscopy Analysis

The CDOM absorption spectra were measured over the 200–800 nm range with a 1 nm increment using a UV–visible spectrophotometer (Shimadzu UV-2550) and a 10 cm quartz cuvette. Ultrapure Milli-Q water was used as the reference. Each sample was scanned three times [37]. The data were corrected to remove the scattering effects and baseline fluctuations by subtracting the value at 700 nm from each spectrum. The CDOM absorption coefficients were obtained using Equation (1) [38]:

(1) a(λ)=2.303×D(λ)/L

where λ is the wavelength, L is the cuvette path length, a(λ) is the absorption coefficient at wavelength λ, and D(λ) is the optical density at wavelength λ.

$S_{g}$ is the exponential slope of the CDOM absorption spectra, which can be determined using Equation (2):

(2) $$a(\lambda )=a(\lambda_{0})e^{S_{g}\left(\lambda_{0}-\lambda \right)}+k$$

where $\lambda_{0}$ is a reference wavelength (nm) (440 nm in this study); and the data were fitted over the range of 300–500 nm. k is an additional background parameter that allows for any baseline shift or attenuation unrelated to the CDOM [39].

#### 2.2.2. Fluorescence Spectroscopy Analysis and PARAFAC Analysis

The fluorescence spectra of the CDOM were measured using a Hitachi F-7000 fluorescence spectrophotometer (Hitachi High-Technologies, Tokyo, Japan). The excitation wavelength was 200–450 nm, with a 5 nm interval. The emission wavelength was 250–600 nm. A 1 nm interval was used to obtain the fluorescence spectra. The three-dimensional fluorescence spectrum of Milli-Q ultrapure water was subtracted to remove the Raman scattering of the pure water. Quinine sulfate (0.01 mg L^−1^) was used for the fluorescence calibration [40,41].

Parallel factor analysis (PARAFAC) was conducted using the DOMFlour toolbox in Matlab2008a. PARAFAC was employed to analyze the fluorescence and compositional properties of the CDOM [42,43].

#### 2.2.3. DOC Measurements

The DOC concentration was measured using a Shimadzu TOC-VCPH total organic carbon analyzer (Shimadzu Co., Japan, temperature: 680 °C) [44]. The high-temperature catalytic oxidation (HTCO) method was used to convert the DOC into CO_2_, which was then quantitatively measured using a nondispersive infrared detector. Each sample was analyzed twice, with a typical deviation of <2%. Then, the DOC concentration was determined from the average value. KHC_8_H_4_O_4_ was used as the carbon standard. Standard ocean water with a known DOC was used as a reference. Instrumental and procedural Milli-Q water blanks were analyzed each day.

#### 2.2.4. Measurements of Chlorophyll-a, Suspended Sediments, and Salinity

The chlorophyll-a (Chl-a) concentration was measured following the standard fluorometric protocol [45]. Each frozen Whatman GF/F filter was extracted using 90% acetone, and the resulting fluorescence was measured using a Turner Designs Fluorometer (Model 10). This instrument was calibrated annually using a commercially available Chl-a standard (Sigma).

The suspended sediments (SS) were measured gravimetrically using preweighed cellulose acetate membrane filters (47 mm diameter, 0.45 μm pore size).

The salinity was measured using a precalibrated conductivity, temperature, depth (CTD) sensor unit (Sea-Bird Electronics, SBE-917 plus).

## 3. Results and Discussion

### 3.1. DOC Distribution

The DOC concentrations ranged from 0.771 to 2.644 mg L^−1^, with an average of 1.172 ± 0.52 mg L^−1^. This is in the same range as those of domestic estuaries, such as the Pearl Estuary [46,47,48], but this range is much lower than those of overseas coastal regions, e.g., the Orinoco River Estuary, the coastal areas of the Southern Baltic Sea, and the Gulf of Mexico estuaries [49,50,51], as shown in Table 1.

**Figure 2:**
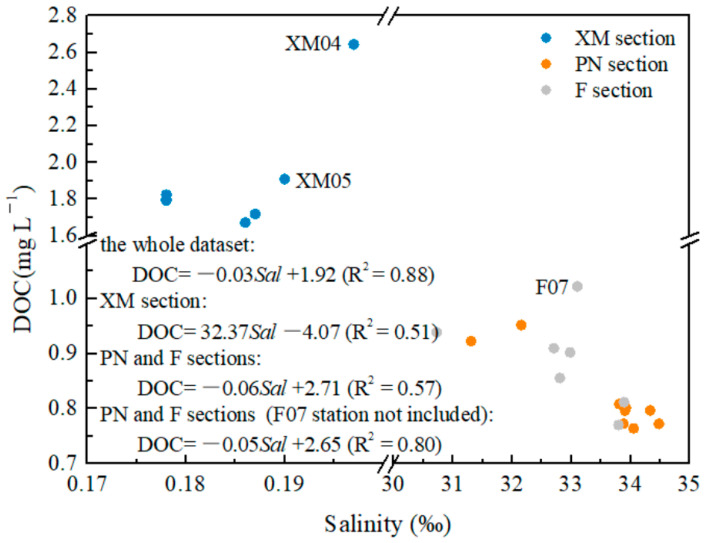
Relationship between the dissolved organic carbon (DOC) and salinity. Distinct positive correlations were observed for the inclusive dataset and the datasets of the respective three sections, even though there was no medium salinity dataset.

Overall, the DOC decreased from the inner estuary to the offshore area (Figure 2). In particular, the DOC values were 0.773–0.952 mg L^−1^ and 0.771–1.022 mg L^−1^ and the salinity values were 30.725–34.48‰ and 30.725–33.891‰ in sections PN and F, respectively. DOC decreased gradually with increasing salinity in both sections, and section F had a slightly higher average DOC (0.887 mg L^−1^) than section PN (0.821 mg L^−1^). As a comparison, the riverine section XM had a very low salinity (average of 0.185‰) and a much higher DOC (average of 1.909 mg L^−1^). Abnormally high DOC was observed at stations XM04 and XM05. Negative correlation between the DOC and salinity was observed in section XM if stations XM04 and XM05 are not considered.

### 3.2. CDOM Absorption Properties

#### 3.2.1. Absorption Spectrum

**Figure 3:**
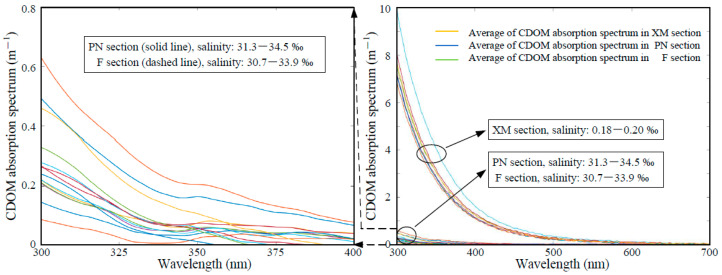
Absorption spectral curves for the chromophoric dissolved organic matter (CDOM) in all three sections. There are distinct differences between the slopes of the dataset for section XM and those of sections PN and F.

The absorption spectra of all of the samples decrease exponentially with increasing wavelength. A distinct blue shift is observed in the wavebands where an obvious decay occurs from 400 nm at station XM01 with a salinity of 0.18‰ to 300 nm at offshore station PN09 with a salinity of 34.5‰. The samples can be divided into two groups based on the slopes of the curves and the wavebands with the maximum attenuation, which is in good agreement with the salinity gradients of the three sections (Figure 3).

#### 3.2.2. a(355)

The absorption coefficient at a specific wavelength λ (e.g., 355, 375, or 440 nm) is usually adopted to quantify the CDOM, since the CDOM component contains a variety of mixtures and its concentration is difficult to measure directly. In this study, a(355) was selected to make our results comparable with other research results (see Table 1). The XM section shows highest a(355) ranging from 2.476–3.742 m^−1^. An extraordinarily high a(355) value (3.742 m^−1^) was observed at station XM04. A significantly low a(355) value was observed in the PN section (average of 0.107 m^−1^ varying from 0.046–0.207 m^−1^) and F section (average of 0.178 m^−1^ varying from 0.115–0.253 m^−1^). The a(355) value was slightly higher in section F than in section PN.

The a(355) value in this study is similar to those reported in previous studies in the Changjiang Estuary, as well as those in the Pearl Estuary, but it is much lower than those of most of the world’s major estuaries (see Table 1).

**Figure 4:**
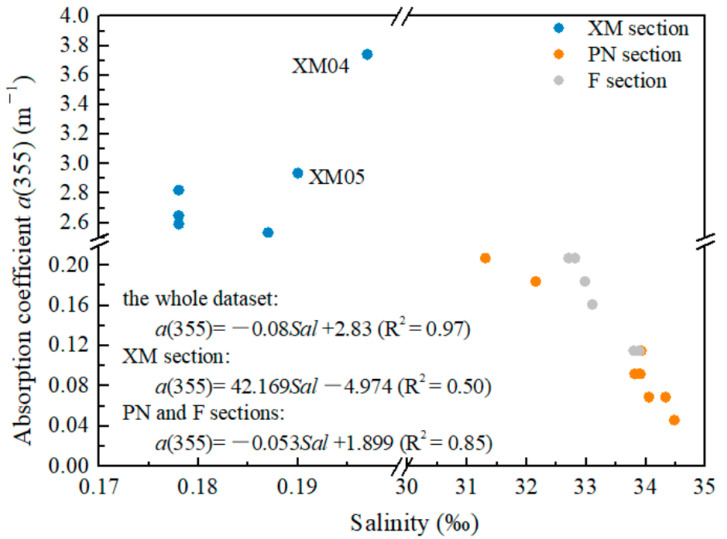
Relationship between salinity and a(355). Strong correlations for the datasets of sections PN and F and for the entire dataset.

We noticed that the a strong negative linear correlation between the salinity and a(355) was observed for both sections PN and F and for the entire dataset (Figure 4). It is noteworthy that abnormally high a(355) values are observed at XM04 and XM05 stations with high DOC.

#### 3.2.3. $S_{g}$

$S_{g}$ has been demonstrated to be useful in distinguishing the composition and sources of the CDOM. The $S_{g}$ depends strongly on the chosen wavelength interval and less strongly on the method used to determine the parameter [39]. Usually, $S_{g}$ is fitted over a range of 300–500 nm, and steeper slopes indicate materials with lower molecular weights or decreasing aromaticity [62]. Most of the CDOM in the river was contributed to by terrestrial materials with higher aromatization, which are resistant to degradation. As a result, the riverine CDOM has a lower $S_{g}$, and the marine water has a higher $S_{g}$.

In this study, an exponential model of the CDOM absorption spectrum was established:

(3) $$a(\lambda )=a(440)e^{0.0213\times (440-\lambda )}$$

$S_{g}$ varied from 0.014 to 0.032 nm^−1^, with a mean of 0.021 nm^−1^. Stable, low $S_{g}$ values were observed along section XM, ranging from 0.0176 to 0.0180 nm^−1^. There were relatively higher $S_{g}$ values along both sections PN and F, with large fluctuations, ranging from 0.0143 to 0.023 nm^−1^ and from 0.025 to 0.0318 nm^−1^, respectively. It should be noted that the $S_{g}$ values in section F were higher than those in section PN.

**Figure 5:**
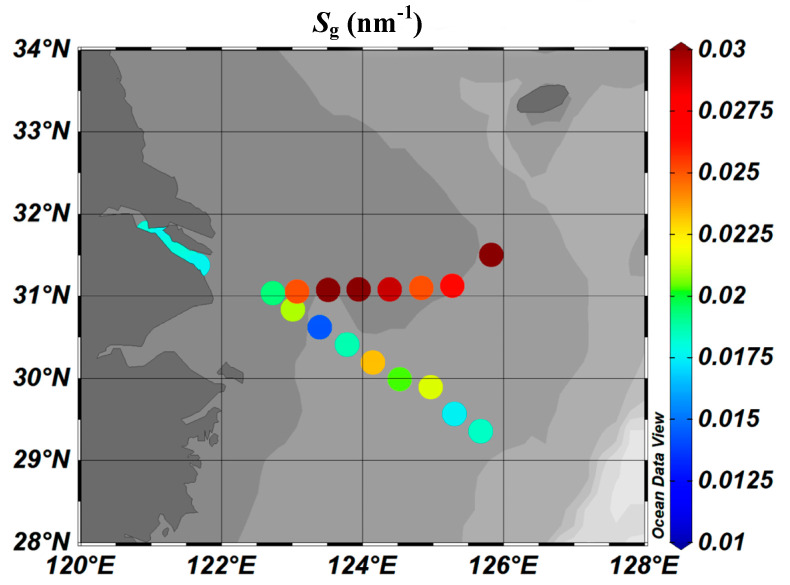
The distributions of the $S_{g}$ values obtained in this study. $S_{g}$ increases with increasing salinity, which is opposite to the correlation between a(355) and salinity (Figure 4).

Overall, the $S_{g}$ increased with increasing distance from shore (Figure 5), and exhibited a trend opposite that of the a(355) and DOC, which is consistent with previous findings (Table 1) [3,63,64].

### 3.3. CDOM Fluorescence Properties

#### 3.3.1. Fluorescence Intensity Fs(355)

**Figure 6:**
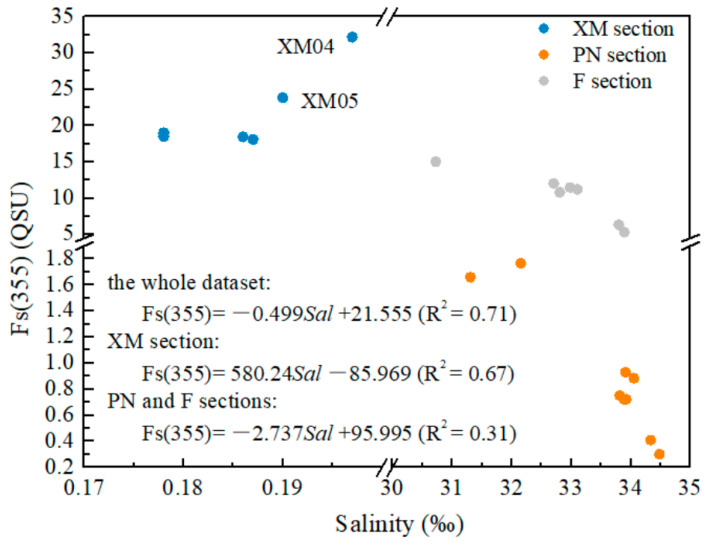
Relationship between salinity and Fs(355) for the datasets of sections PN and F and for the entire dataset.

The total fluorescence intensity of the CDOM in this study was characterized by the fluorescence intensity of Fs(355) at an excitation wavelength of 355 nm with emission spectrum recorded at 450 nm (Ex/Em = 355/450 nm) [48,58,61]. The Fs(355) values of sections PN and F were 0.301–1.767 quinine sulfate units (QSU) (average of 0.905 QSU) and 1.45–2.01 QSU (average of 1.39 QSU), respectively, and there was a decreasing trend along both sections. In contrast, section XM had the highest average Fs(355) value (21.294 QSU), with values ranging from 18.107–32.181 QSU. Stations XM04 and XM05 had abnormally high Fs(355) values. A strong correlation between Fs(355) and salinity was observed for the whole dataset and for PN and F sections, respectively (Figure 6).

**Figure 7:**
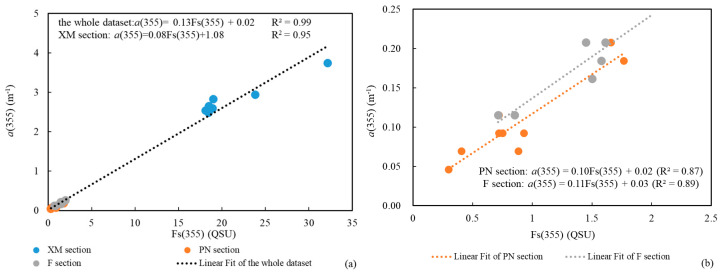
Relationship between Fs(355) and a(355). (**a**) Entire dataset for all three sections; (**b**): dataset for sections PN and F. Strong positive correlations between Fs(355) and a(355) were found in sections PN and F.

Moreover, Fs(355) exhibited similar distribution patterns to a(355) along sections PN and F, and along XM section as well (Figure 7), which was consistent with the results of previous studies [2,65].

#### 3.3.2. CDOM Excitation–Emission Matrix Spectroscopy (EEMS)

**Figure 8:**
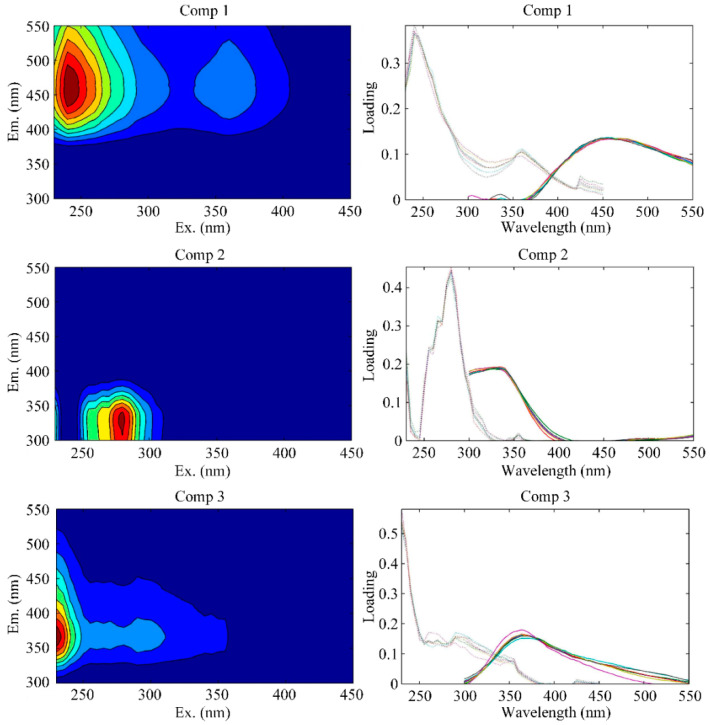
Excitation–emission matrix fluorescence spectroscopy (EEMS) contour plots and loadings of each component determined using parallel factor analysis (PARAFAC). Three fluorescence components, including the humic-like component (Comp1), the protein-like component (Comp2), and the protein-like component (Comp3), were extracted using PARAFAC.

Three fluorescence components, including C1 (humic-like component), C2 (protein-like component), and C3 (protein-like component), were extracted from all 23 samples using PARAFAC (Figure 8, Table 2), which is similar to the method used in previous studies conducted in this area [66]. C1 has a maximum excitation/emission at 240/456 nm. This is similar to the traditionally defined humic-like fluorescence peak A ((230–260)/(380–460) nm), which primarily originates from terrestrial sources. C2 has a maximum excitation/emission at 280/328 nm, which was confirmed to be tryptophan-like fluorescence peak T ((270–280)/(320–350) nm). Tryptophan-like peak T can be derived from both allochthonous and autochthonous sources [67,68]. C3 has a maximum excitation/emission at 230/366 nm, resembling a combination of peak N (280/370 nm) and peak T. Peak N is believed to represent labile materials produced as a result of biological production [69,70].

#### 3.3.3. Fluorescent Components

**Figure 9:**
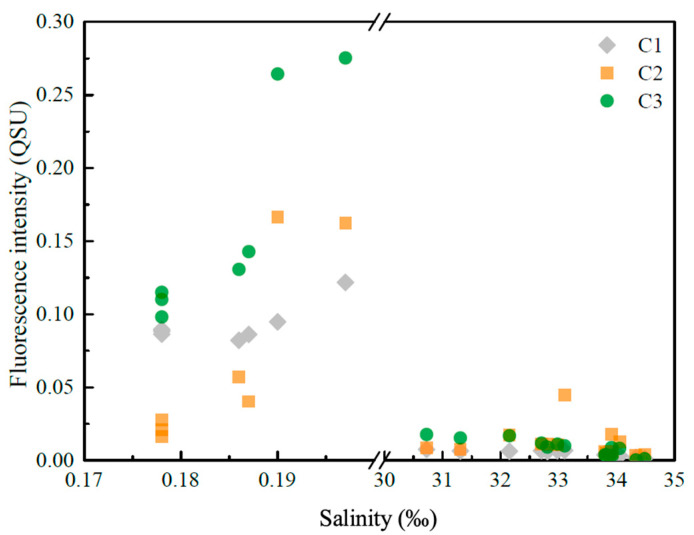
Variations in the fluorescence intensities of C1, C2, and C3 at the different stations. In general, the fluorescence intensities of all of the CDOM components decreased from the inner estuary to the offshore area.

As is shown in Figure 9, the fluorescence intensities of components C1, C2, and C3 were much lower along sections PN and F than along section XM.

Along sections PN and F, all three components exhibited similar patterns: decreasing with increasing salinity. It should be noted that the intensity of C1 was relatively steady throughout section XM, whereas C2 and C3 varied consistently, and both increased abruptly at stations XM04 and XM05.

## 4. Discussion

Relationships between the DOC and the optical properties of the CDOM and the possible impact factors in different sections were analyzed in this study. The analysis of variance (ANOVA) statistics were utilized to determine whether the changes in the DOM composition were statistically significant. The F values of the one-way ANOVA of the Fs(355) (F > 70.67, *p* = 8.57 × 10^−10^), DOC (F > 73.68, *p* = 5.94 × 10^−10^), and a(355) (F > 295.75, *p* = 1.4 × 10^−15^) values are all greater than 1, indicating that the differences in the three sections were statistically significant. The following discussion is based on the results of the ANOVA statistical analysis.

### 4.1. Relationship between CDOM and DOC

**Figure 10:**
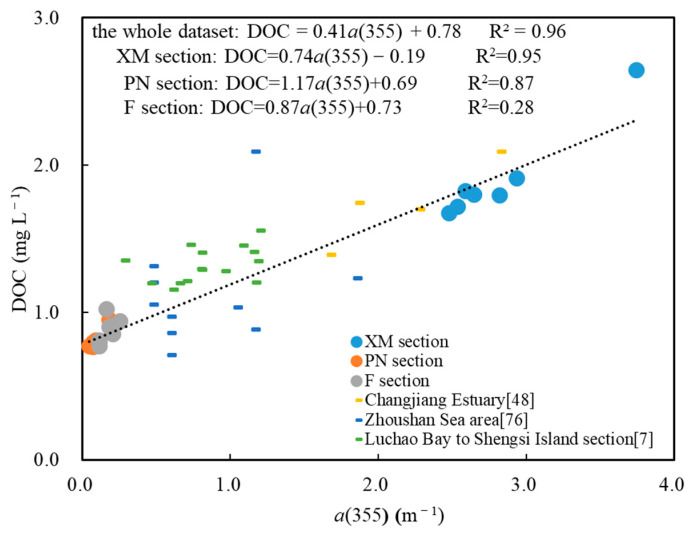
The correlations between the a(355) and dissolved organic carbon (DOC) in the three sections sampled in this study. The datasets from previous studies [7,48,76] are distributed evenly along the regression line for this study.

The low production of soil organic matter due to the low vegetation coverage in the Changjiang Basin is the most likely reason for the low DOC in Changjiang Estuary. In addition, the high concentrations of suspended particles in the Changjiang River and in the estuary are another important mechanism for the removal of DOC through adsorption [75]. It is notable that the low DOC is consistent with the low a(355). Strong correlations between the a(355) and DOC were observed in sections PN and XM. However, a weaker positive correlation was observed in section F (Figure 10).

**Figure 11:**
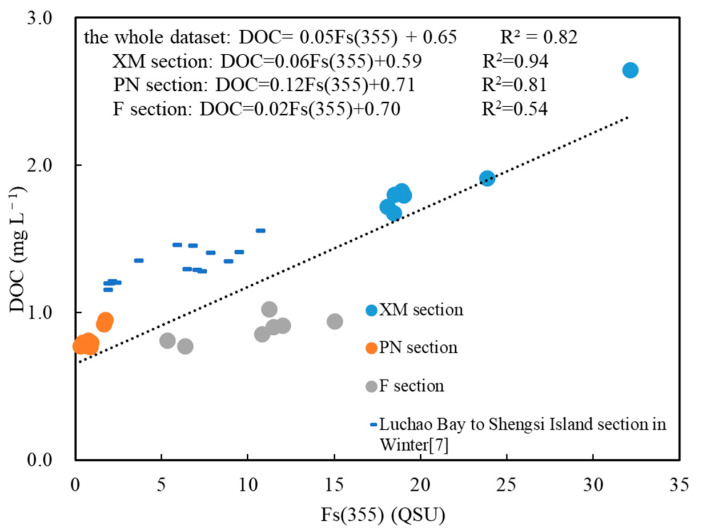
The correlations between the Fs(355) and dissolved organic carbon (DOC) in the three sections sampled in this study. The dataset from a previous study [7] is distributed evenly along the regression line for this study.

Fs(355) shows strong correlations with DOC for the whole dataset and for the three sections respectively (Figure 11). Particularly, the correlation between the Fs(355) and DOC in F section was better than that between the a(355) and DOC, but it was still lower than those in sections PN and XM (Figure 10). The weaker correlations between the a(355), Fs(355), and DOC in section F may be due to the more complicated material composition supplied by multiple sources and to the different abilities of a(355) and Fs(355) to represent the diverse DOC species [76]; however, further investigation is needed to clarify this issue. Usually, terrestrial CDOM absorbs light strongly in the ultraviolet band due to the tannins and lignin constituents [77]; whereas marine CDOM has few aromatic rings, and its ability to absorb light is weak in the 355 nm band [78,79]. Thus, the a(355) value has advantages in terms of reflecting terrestrial CDOM [80]. Additionally, it should be noted that with the abnormally high DOC, a(355) and Fs(355) at station XM04 and XM05 will impact the reliability of their correlations.

**Figure 12:**
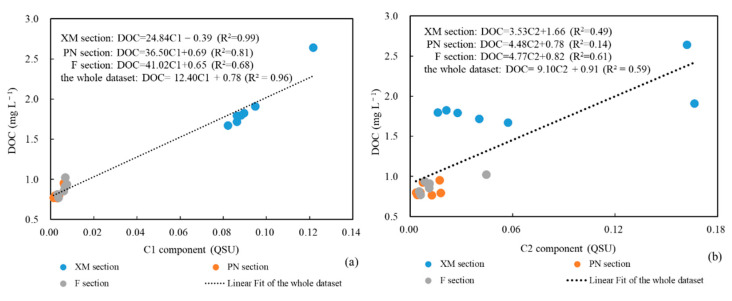

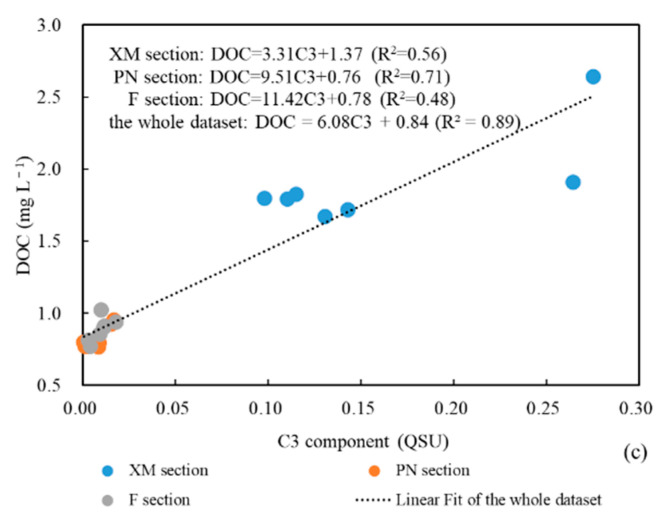
Correlations between the dissolved organic carbon (DOC) and the chromophoric dissolved organic matter (CDOM) fluorescence intensities of the components: (**a**) C1 versus DOC; (**b**) C2 versus DOC; and (**c**) C3 versus DOC. The correlations between the DOC and the fluorescence intensities of the three fluorophores in the three different sections differ.

The correlations between the fluorophores and the DOC were further analyzed to explore the potential ability of the fluorescence intensities of the different fluorophores to represent the DOC content and species. In this study, the correlations between the DOC and the fluorescence intensities of the three fluorophores differed in the three different sections (Figure 12). (1) There was a strong correlation between C1 and the DOC for the entire dataset and in each section. The correlation coefficients along of the three sections decreases in the following order: XM > PN > F. Since C1 represents the humic-like component, the strong correlation between C1 and the DOC found in this study indicates the dominant control of the terrestrial materials in the study area and the decreasing impact of the terrestrial materials along the three sections. (2) The correlations between the protein-like components (C2 and C3) and the DOC vary along the three sections. A stronger positive correlation between C3 and the DOC was observed along section PN, which may indicate that biological production is one of the most important components of the DOC along section PN [69,70]. However, C2 and the DOC exhibit a stronger correlation along section F due to the more abundant input of bacteria-derived DOM and interstitial water through resuspension [80].

### 4.2. DOC Source Analysis

To obtain deeper insights into how the variable DOC component affects the relationships between the CDOM and the DOC, the possible DOC material sources that may introduce extra DOC and change the main DOC constituents along the three different sections were investigated.

**Figure 13:**
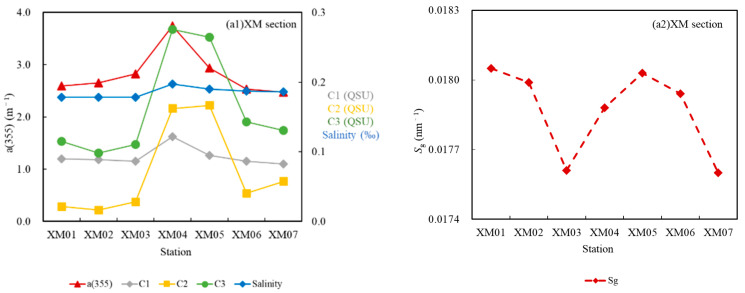

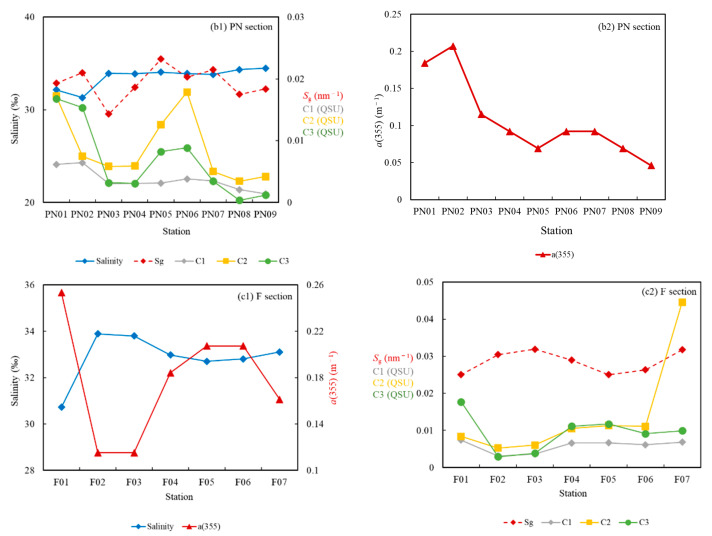
a(355) (m^−1^), $S_{g}$ (nm^−1^), salinity (‰), and the fluorescence intensity of the fluorophores (QSU) along the three sections. (**a1**,**a2**) section XM; (**b1**,**b2**) section PN; and (**c1**,**c2**) section F.

Particularly, Section XM is dominated by terrigenous materials, and therefore, it has the highest DOC concentration and a(355), Fs(355), and C1 values and the lowest $S_{g}$ values [53,80]. However, the abnormal increases in the DOC, a(355), Fs(355), C2, and C3 observed at stations XM04 and XM05 suggest sewage input from the Huangpu River (Figure 13(a1,a2)). Due to the rapid development of industry and agriculture in recent years, the water quality of the Huangpu River, which is an important tributary of the Changjiang Estuary, has deteriorated, and contains a great deal of organic matter, leading to the intensified autochthonous production of DOM [81,82]. This polluted water could have migrated to stations XM05 and XM04 or even further upstream after it flowed into the Changjiang River during high tides. The abnormally high DOC and CDOM optical properties due to the input from Huangpujiang River should be cautious, which may introduce overestimated DOC if they are used for DOC retrieval models’ construction.

Usually, C3 is associated with biological production [83], but the covariation in C3 and C2 along section XM mostly suggests that C3 contains tryptophan-like peak T (derived from an allochthonous source), which was noted by Hong et al. [84] in the Jiulong River watershed, and has been verified by its correlation with the tryptophan-like component.

Along section PN (Figure 13(b1,b2)), the a(355) and C1 vary consistently with the DOC, suggesting the predominance of the terrestrial materials carried by the CDW and that the diffusion of the CDW is the basic hydrodynamic process. However, C2 and C3 vary differently than C1. They decrease gradually (similar to C1) at the first four stations, but they increase unexpectedly at stations PN05 and PN06. This is accompanied by abnormal increases in the Chl-a concentration from 0.75 μg L^−1^ at station PN04 to 1.29 μg L^−1^ at station PN05 and to 1.09 μg L^−1^ at station PN06. Because they are protein-like fluorescence components, C2 and C3 are usually attributed to autochthonous production during the exponential growth of phytoplankton [67]. Usually, high chlorophyll-a concentrations are observed in the Changjiang Estuary, especially in spring when the TWC converges with the saline water on the continental shelf [85], which promotes the aggregation of marine autochthonous substances. Thereafter, in addition to the terrestrial materials carried by the CDW, autochthonous DOM is also a main source of the DOC along section PN. The regional occurrence of marine autochthonous substances could be one of the main factors impairing the conservative behavior of the DOC along section PN.

Along section F (Figure 13(c1,c2)), C1 continuously decreases from station F01 to station F03, and it abruptly increases at station F04. Station F04 is located in the southwestern part of a cyclonic eddy to the southwest of Jeju Island, where materials from both the Changjiang Basin and the Old Huanghe Delta settle to the bottom [86]. This unexpected sustained increase in C1 at certain stations may suggest the input of additional terrestrial materials at station F04, except for the materials from the Changjiang Basin carried by the CDW.

Similar to C1, C2 and C3 decrease at the first few stations and then gradually increase starting at station F04. This may suggest the mitigation of the influence of the protein-like component (peak T) carried by the CDW runoff and the enhancement of the impact of the autochthonous marine materials, which are stimulated by the convergence of the SCC and the CDW.

An unexpected maximum for component value of C2 was observed at station F07, which is consistent with its abnormally high DOC concentration. Station F07 is located in an area where complex currents exchange materials and mix, the rich nutrients support plankton blooms, and the biological activities and degradation of biological debris produce protein-like fluorescence peaks [87]. Furthermore, there is an upwelling system to the south of Jeju Island (125°30′ E–127° E), which contains rich nutrients, and the frequent resuspension of bottom sediments has been observed [88]. The release of sediment interstitial water and/or sediment resuspension could cause the higher fluorescence intensity of the tryptophan and tyrosine in the bottom water [87], thus inducing an increase in the intensity of the protein-like C2 and the DOC in the surface sea water during upwelling. However, an abrupt decrease in the a(355) value was observed at this station, suggesting the weakness of using a(355) to represent marine CDOM and the weak influence of the terrestrial DOC.

## 5. Conclusions

In this study, the correlations between the optical properties of the CDOM and the DOC were discussed for the whole dataset, the south branch of Changjiang River (XM section), the main axis and the northern boundary of the CDW, i.e., sections PN and F, respectively. Both the absorptive and fluorescent properties of the CDOM in these sections and the possible sources of the extra DOC input were analyzed. The ability of the optical properties of the CDOM to reflect the main intrinsic mechanism impairing the correlation between the optical properties of the DOC and the CDOM in the CDW were investigated, and it was found that in addition to the hydrodynamic environment, phytoplankton, and microbial activities, the material sources may have a profound impact on the spatial CDOM and DOC variations and their correlations. Specifically, the following conclusions were drawn based on the results of this study.

Essentially, DOC shows conservative behavior, and the dilution of terrestrial material transported by CDW is dominant, controlling the DOC distribution features and the CDOM optical properties correspondingly. No obvious removal of DOC was observed in the three sections, however, extra inputs were observed in all three sections in different ways, impacting the correlations between the CDOM and DOC. In particular, the polluted water input from Huangpujiang River will produce overestimated DOC if data are used without cautious screening. The sustained terrestrial material input in section F included terrestrial materials from the Huanghe Basin, mainly from the Old Huanghe River Delta transported by the SCC, and from the Yellow Sea transported by the YSWCC, besides from the Changjiang Basin transported by the CDW. This may be one of the main factors causing the differences in the correlations between the optical properties of the DOC and CDOM. However, the degree of impact from the continuous material input from Huanghe River Basin should be studied further. In the PN section, the impact of autochthonous DOC input on the correlation between DOC and CDOM can be neglected in comparison with the conservative dilution procedure on the main axis of CDW.

Both a(355) and Fs(355) were demonstrated to be promising indexes for reflecting the DOC. However, it should be noted that a(355) worked well for the datasets, except for section F, while Fs(355) exhibited a strong correlation with the DOC in section F. Fs(355) was demonstrated to be a good indicator of the DOC concentrations of multiple source materials. Moreover, compared with a(355) and Fs(355), the fluorescence components were demonstrated to be powerful indicators of the different DOC species in complex estuary environments.

This study revealed that for the introduction of different species of DOC in multiple ways and for the different abilities of the CDOM absorptive and fluorescence properties to reflect the DOC, there are significant differences in the relationships between the DOC and the optical properties of the CDOM, even within the CDW. To establish a feasible inversion algorithm for determining DOC from the optical properties of the CDOM, long-term observations of the seasonal and annual variations in the DOC inputs from the different sources in this area are necessary in order to consider the effects of the CDW discharge, the magnitude of the currents, and even the wind stress. The optical properties of the DOC input from the different sources also require further study, and chemical analysis of different DOC species is a solution to provide insights into the intrinsic mechanisms determining the variable optical properties of the CDOM.

## Figures and Tables

**Table 1:** Comparison of the a(355) and $S_{g}$ values of the chromophoric dissolved organic matter (CDOM) from several estuaries around the world.

Research Areas		$a(355)\;(m^{-1})$	$S_{g}\;({\mathrm{nm}}^{-1})$	Salinity (‰)	References
Changjiang Estuary (summer)		0.1–3.2	0.017–0.020 (300–650 nm)	0–32.0	[52]
Changjiang Estuary (spring)		1.152–8.715	0.0034–0.014 (380–800 nm)	/	[53]
Changjiang Estuary (summer)		0.20–0.73	/	0.2–25.3	[54]
Changjiang Estuary (summer)		0.20–0.77	/	0.3–29.5	
Changjiang Estuary (spring)		0.10–2.82	0.017–0.020 (300–500 nm)	0.12–29.4	[3]
Changjiang Estuary (winter)		0.11–1.20	0.008–0.018 (275–295 nm)	18.7–34.9	[8]
Changjiang Estuary (summer)		0.23–1.91	0.012–0.025 (275–295 nm)	4.0–33.6	
Pearl River Estuary (November)		0.24–1.93	0.0138–0.018 (300–500 nm)	0–32.49	[55]
Pearl River Estuary (June)		0.34–1.40	/	0–34.96	[47]
South of the North Sea (February)	Scheldt Estuary	0.97–4.30 a(375)	0.0167–0.019 (350–500 nm)	0.7–29.6	[56]
	Belgium coastal sea	0.20–1.31 a(375)	0.0110–0.020 (350–500 nm)	29.8–33.6	
Chesapeake Bay	River end member	2.2–4.1	0.0163–0.019 (280–650 nm)	0–35.0	[57]
	coast	0.4–1.1	0.0178–0.022 (280–650 nm)		
Mississippi Estuary (summer)		1.2–4.2	/	/	[49]
Amazon Estuary (winter)		0.14–3.12	/	/	[58]
Georgia coast		0.06–1.20	/	/	[59]
Northern Gulf of Mexico		3.96–17.52 a(350)	/	0–37.0	[60]
Southern Beaufort Sea		0.018–1.08 a(440)	0.015–0.023 (350–500 nm)	0–35.0	[61]
Section XM (August 2011)		2.476–3.742	0.0176–0.018	0.18–0.20	This study
Section PN (March 2011)		0.046–0.207	0.0143–0.023	31.31–34.5	
Section F (March 2011)		0.115–0.253	0.025–0.0318	30.72–33.9	

Note: a(355) and a(375) represent the CDOM absorption coefficients at 355 nm and 375 nm, respectively.

**Table 2:** Characteristics of the chromophoric dissolved organic matter (CDOM) components determined using the parallel factor analysis (PARAFAC) model in this study and comparison with the results of previous studies.

	Ex/Em (nm)	Coble [71,72] (Ex/Em (nm))	References (Ex/Em (nm))
C1	240/456	peak A: 230–260/380–460; humic-like component	C3: 270 (360)/478 [69]
			C4: 250 (360)/440 [67]
			C8: 250 (380)/416 [72]
			C1: 270 (365)/453 [40]
			C1: ≤250 (335)/428 [73]
C2	280/328	peak T: 270–280/320–350; protein-like component	C5: 280(240)/368 [69]
			C7: 280/344 [67]
			C7: 240 (300)/338 [72]
			C4: 280/318 [68]
			C6: 250(290)/356 [74]
			C4: 275/328 [73]
C3	230/366	combination of peak N (280/370) and peak T (270–280/320–350); protein-like component	C5:280(<240)/368 [69]
			C4: 250 (320)/370 [72]
			C5: 285/362 [68]
			C2: ≤250(300)/368 [73]

## Data Availability

The data contained in this paper are available from the authors.

## Abbreviations

CDOM	Chromophoric Dissolved Organic Matter
CDW	Changjiang Diluted Water
chlorophyll-a	Chl-a
DOC	Dissolved Organic Carbon
DOM	Dissolved Organic Matter
ECS	East China Sea
EEMs	Excitation Emission Matrix Fluorescence spectroscopy
HTCO	High-Temperature Catalytic Oxidation
MTZ	Maximum Turbidity Zone
PARAFAC	Parallel Factor Analysis
SCC	Subei Coastal Current
SS	Suspended Sediments
TWC	Taiwan Warm Current
YSWC	Yellow Sea Warm Current
YSWCC	Yellow Sea Western Coastal Current
ZCC	Zhejiang Coastal Current
